# Biological characterization of multidrug-resistant *Pseudomonas aeruginosa* phage PaeP_Ls and its efficacy in septic mice

**DOI:** 10.1128/spectrum.02050-25

**Published:** 2026-01-07

**Authors:** Shuhong Luo, Li He, Ling Wu, Yong Zhang, Lin Ning, Juan Chen, Jian Hu, Jiajiao Liu, Qin Wang, Yujie Bai, Jian Feng, Fuxiang Li

**Affiliations:** 1 Department of Respiratory and Critical Care Medicine, The First People’s Hospital of Shuangliu District, Chengdu, China; 2 Department of Nutrition, The General Hospital of Western Theater Command, Chengdu, China; 3 Department of Critical Care Medicine, The General Hospital of Western Theater Command, Chengdu, China; National Microbiology Laboratory, Winnipeg, Manitoba, Canada

**Keywords:** *Pseudomonas aeruginosa*, phage, genetic analysis, phage-resistant bacterial variant, sepsis, phage therapy

## Abstract

With the widespread use of antibiotics, multidrug-resistant *Pseudomonas aeruginosa* (MRPA) is increasing year by year. Phage therapy is gaining more and more attention as an alternative therapy to antibiotics. The main objective of this study was to assess the efficacy of PaeP_Ls phage against MRPA-induced sepsis in mice. We isolated and purified a lytic *P. aeruginosa* phage PaeP_Ls. PaeP_Ls had an optimal multiplicity of infection (MOI) of 0.001 under our conditions, latent and lytic periods of 25 and 70 min, and a burst size of about 50 plaque-forming units (PFU) per infected cell. Its lysis rate against MRPA was 53.33%. PaeP_Ls was a double-stranded DNA virus with a genome length of 45,217 bp and GC content of 52.46%, encoding 81 coding sequences and three tRNAs, without drug resistance genes or virulence factors. We used PaeP_Ls to treat the *P. aeruginosa* strain MRPA1-infected septic mice and found that intraperitoneal injection of high concentrations of PaeP_Ls (MOI = 10) significantly increased the survival rate of the mice (*P* < 0.0001). Intraperitoneal injection of (5 × 10^11^ PFU/mouse) phage in septic mice caused a significant reduction in the bacterial load in the blood of the mice. Histopathology showed that inflammatory cell infiltration and congestive edema were significantly reduced in the liver, lungs, and kidneys in the phage treatment group. PaeP_Ls shows therapeutic potential against the MRPA1 strain of *P. aeruginosa*.

**IMPORTANCE:**

The lytic bacteriophage PaeP_Ls was isolated and thoroughly described in this study; its genomic analysis showed no virulence or resistance threats, indicating exceptional safety. More importantly, we showed that PaeP_Ls therapy effectively removed pathogens from the bloodstream, reduced organ damage, and greatly increased survival rates in a therapeutically relevant mouse sepsis model. This increases the translational development of phage therapy toward clinical application in addition to offering a novel phage option for fighting multidrug-resistant *Pseudomonas aeruginosa* infections.

## INTRODUCTION

*Pseudomonas aeruginosa* is an aerobic non-fermenting gram-negative bacterium that causes hospital-acquired infections, respiratory-associated pneumonia, localized purulent inflammation, keratitis, and otitis media (1). As one of the most frequent opportunistic pathogens causing nosocomial infections (2, 3), *P. aeruginosa* hardly affects healthy individuals but causes high morbidity and mortality in ventilator-associated pneumonia, bacteremia, and immunocompromised patients (4). Due to the overuse of antibiotics, *P. aeruginosa* strains resistant to multiple antibiotics—such as aminoglycosides, quinolones, and β-lactams—are becoming increasingly prevalent (5). Furthermore, *P. aeruginosa* possesses intrinsic and acquired resistance to many classes of antibiotics, with the production of β-lactamases and aminoglycoside-modifying enzymes being its most important resistance mechanisms. The accumulation of multiple resistance mechanisms has eventually led to increased multi-resistant and even “pan-resistant” strains worldwide (6, 7). Certain extensively resistant clones, such as ST175, ST111, and ST235, are often associated with specific resistance mechanisms (8). According to 2019 data, *P. aeruginosa* is the sixth pathogen among bacteria with the highest number of deaths due to antimicrobial resistance (9). Carbapenem-resistant *P. aeruginosa* has been categorized by the WHO as a “high priority” group of pathogens for which novel therapeutic options are urgently needed (10).

With the recent surge in antibiotic resistance, there has been a resurgence of interest in bacteriophage therapy (11). The advantages of phage therapy include a high specificity of most phages, low natural toxicity, and anti-biofilm activity of phages (12). Unfortunately, an obvious limitation of phage therapy is the overwhelming evidence that bacteria may rapidly evolve resistance to phage infections, which may compromise its efficacy (13). However, phage therapies are usually supposed to be doubly effective by lysing the target bacteria and making them more sensitive to antibiotics when they develop phage resistance (14). The potential promise of phage therapy as an effective antimicrobial strategy has been demonstrated by the increasing number of successful cases of phage therapy used for the treatment of clinical multidrug-resistant *P. aeruginosa* (MRPA) infections in recent years (15, 16).

The narrow host spectrum of phages necessitates the continuous isolation and characterization of new phages to meet the demand for therapeutic phages. In this study, we isolated and characterized a lytic *P. aeruginosa* phage, PaeP_Ls, and evaluated its therapeutic efficacy against septic mouse infections, providing preclinical evidence for the feasibility of phage therapy.

## MATERIALS AND METHODS

### Bacterial strains

MRPA strains (MRPA1-16) were provided by the Department of Laboratory Medicine of the General Hospital Western Theater Command and identified by the VITEK2-compact system. It should be noted that this set of strains serves as a convenient sample for preliminary assessment of the phage lysis spectrum; detailed clinical origins and complete molecular typing information were not included in this study. Second-generation gene sequencing of MRPA1 revealed the presence of multiple resistance genes such as adeL, macB, evgS, NmcR, and OprM, which are resistant to a variety of antibiotics, including carbapenems, fluoroquinolones, and β-lactams. The strain type of MRPA1 is ST282, which is not a globally prevalent drug-resistant ST type and can be isolated from southern India and some intensive care unit wards, where extensive resistance to β-lactams has been observed in clinical isolates (17, 18). The minimum inhibitory concentration (MIC) of antibiotics was determined according to the Clinical and Laboratory Standards Institute (CLSI 2018) drug sensitivity test implementation standard.

### Isolation and purification of phage

PaeP_Ls (Pae refers to *P. aeruginosa*, P refers to short-tailed phage [Podovirus], and Ls is the abbreviation of lytic strain) was isolated from unsterilized sewage in the General Hospital Western Theater Command wastewater treatment station. A mixture of 50 mL of 2× concentrated Luria-Bertani (LB) broth and 50 mL of untreated effluent was inoculated with 200 μL of logarithmic growth stage host bacterium MRA1 to enrich with phage. The solution was mixed and incubated overnight in a shaker (37°C, 200 r/min). After centrifuging the culture solution (4°C, 10,000 × *g*, 10 min), the supernatant was filtered through a 0.22 μm filter, and the filtrate was stored at 4°C. The phage was purified using a double agar plate method (in which the concentration of the top layer of agar was 0.7%, and the concentration of the bottom layer of agar was 1.5%) (19). A single plaque was taken with a pipette tip, mixed with the host bacteria, and incubated overnight in a shaker (37°C, 200 r/min). The supernatant was filtered after centrifugation, and the above steps were repeated 3–5 times, resulting in the purified phage PaeP_Ls. The phage titer was determined by plating 100 µL of phages with the standard double-layer plate method. For counting, plates with 30–300 plaque were chosen, and the phage titer (PFU/mL) = number of plaque × dilution times × 10 (20).

Phage particles were concentrated by polyethylene glycol 8000 (PEG 8000) (21). Briefly, the phage solution was treated with NaCl (final concentration, 1 M) and polyethylene glycol 8000 (final concentration, 10%) and incubated for 12 h at 4°C. The concentrated phage suspension was obtained by placing it in a centrifuge, 12,000 × *g*, 4°C, centrifuging for 1 h to collect the concentrated phage and resuspending it in 1 mL of sterile sodium magnesium (SM) buffer.

The phage pellet was purified by CsCl density gradient centrifugation with reference to the method of Luong et al. (22). Briefly, 1 mL of CsCl with densities of 1.30 g/mL, 1.50 g/mL, and 1.70 g/mL was carefully layered in a microcentrifuge tube, respectively, and 9 mL of crude phage suspension was added to the top of the gradient layer, SM buffer was added, and the tube was leveled, placed in an ultra-high-speed centrifuge at 120,000 × *g*, 4°C, centrifuged for 2–3 h, and finally, extracted the purified phage pellet from the bottom of the visible white/gray band using a syringe needle and dialyzed to remove excess CsCl.

### Transmission electron microscopy

Briefly, 20 μL of concentrated phage solution (1 × 10^11^ PFU/mL) was incubated on a carbon-coated copper grid for 2 min and stained with 10 μL of 2% phosphotungstic acid for 90 s. Excess dye was aspirated with filter paper, and the morphology of the phage PaeP_Ls was observed with a transmission electron microscope (TEM, model: JEM-2100Plus, Japan Electronics) at 80 kV.

### Phage host range assay

Spot test assay: 200 μL of the bacterial solution to be tested in the logarithmic growth phase was mixed with 5 mL of semi-solid LB and poured onto solid LB, and 5 μL of purified phage solution (10^8^ PFU/mL) was dropped in the center of the double-layer agar medium and then inverted and incubated at 37°C overnight after being absorbed.

### Temperature and pH stability

The thermal stability testing was performed by incubating the phages (10^8^ PFU/mL) at different temperatures (10°C, 20°C, 30°C, 40°C, 50°C, 60°C, and 70°C), and the aliquots were collected at 20, 40, and 60 min after incubation. Meanwhile, pH stability was tested by diluting the phages (10^8^ PFU/mL) 10-fold with SM buffer at different pH values of 1–13 and incubating for 2 h at room temperature. The phage titer for thermal and pH stability testing was determined using the double-layer agar plate method, and all experiments were repeated three times.

### Determination of the optimal multiplicity of infection

The optimal multiplicity of infection (MOI) of PaeP_Ls was determined as previously described with some modifications (23). Briefly, serial dilutions of MRPA1 were added to aliquots of PaeP_Ls (10^8^ PFU/mL). Mixtures of 200 μL of different MOIs (0.001, 0.01, 0.1, 1, 10, and 100) were added to 5 mL LB medium and incubated with shaking (220 r/min) at 37°C for 3–5 h. Then, the mixture was centrifuged at 12,000 × *g* for 10 min at 4°C to remove residual bacterial cells. The supernatant was filtered through a 0.22 µm filter. The experiments were repeated three times.

### One-step growth curve

The one-step growth curve of PaeP_Ls was determined by mixing it with MRPA1 at an optimal infection complex (MOI = 0.001) under our conditions. The solution was added to a 5 mL LB medium and incubated at 37°C for 15 min. The supernatant was removed after centrifugation at 13,000 × *g* and 4°C for 30 s. The precipitate was resuspended twice with LB medium, and the mixture was added to 5 mL of LB medium pre-warmed at 37°C and incubated for 120 min at 37°C with shaking at 220 r/min. Sample every 10 min at 0, 10, 20, 30, 40, 50, 60, and up to 120 min. The experiment was repeated three times.

### Measurement of adsorption experiments

The mixture of PaeP_Ls and MRPA1 at an MOI of 0.01 was incubated at 37°C, and 100 µL was taken at 0, 2, 4, 6, 8, 10, 12, 14, 16, 18, and 20 min of incubation and mixed with 900 μL SM buffer. The mixture was immediately centrifuged at 13,000 × *g* and 4°C for 1 min, and the phage titer in the supernatant was measured using the double-layer plate method. The phage adsorption rate was the percentage of free phage titer relative to the total phage titer (i.e., initial phage titer) at each time point (24). The experiment was repeated three times.

### Suppression of *in vitro* bacterial growth by phage

The ability of PaeP_Ls to inhibit bacterial growth was determined using optical densitometry (25). Briefly, 100 μL of phage and 100 μL of bacteria were mixed in 96-well plates at MOIs of 10, 1, 0.1, 0.01, and 0.001. The plates were incubated at 37°C for 24 h, and the OD_600 nm_ was measured every 10 min using a High-throughput growth curve analyzer (BMGL abtech, Germany). The experiments were repeated three times.

### Phage re-sensitizes host bacteria to antibiotics

To evaluate the change in MRPA1 resistance, the phage solution was added to the overnight bacterial broth, and then the cultures were grown at 37°C with shaking at 220 r/min. When the culture solution appeared to change from clear to turbid again, the bacterial broth was inoculated onto solid LB plates and incubated overnight at 37°C. The next day, individual colonies were chosen and sent to our hospital’s bacteriology lab for bacterial identification and resistance testing. We used the broth microdilution method recommended by the CLSI to quantitatively determine the MIC and compare changes in bacterial resistance patterns.

### DNA extraction and whole genome sequence analysis

The Proteinase K/SDS method (26) was used to extract phage DNA. Whole genome sequencing libraries were constructed from extracted PaeP_Ls DNA using TruSeqTM DNA Sample Prep Kit-Set A (Illumina, USA) and PE150 sequencing of the prepared libraries using the DNBSEQ-T7 platform. The prepared libraries were sequenced using the DNBSEQ-T7 platform in PE150 mode. Raw sequencing data underwent filtering and quality control with FastQC to obtain clean reads. Subsequently, high-quality data were *de novo* assembled using SPAdes software to yield the complete phage genome. The whole genome of the phage was annotated with RAST (http://rast.nmpdr.org/), and the online BLAST tool (http://www.ncbi.nlm.nih.gov/BLAST) was used for comparative analysis and prediction of genome function. Putative antibiotic resistance genes and virulence factors were screened using the online antibiotic resistance gene and virulence factor libraries (https://cge.food.dtu.dk/services/ResFinder/ and https://cge.food.dtu.dk/services/VirulenceFinder/). The tRNAscan-SE v.2.0 software was used to identify tRNA coding sequences (CDSs), and whole genome mapping was performed using the CGView Server software (http://cgview.ca/). The MEGA 11.0 software was used to construct the phylogenetic evolutionary tree of tail fiber protein and terminal enzyme large subunit genes using the maximum likelihood method, and a bootstrap test was performed 1000 times. The Easyfig 2.2.3 software was used for comparative genetic analysis. The sequencing data are available in GenBank (accession number OP342787).

### Phage therapy in a mouse sepsis model

The safety of phages was assessed by intraperitoneally injecting 10 randomly selected mice with phage (1.0 × 10^11^ PFU/mouse). The clinical symptoms of the mice were observed within 5 days, and the survival rate was calculated.

The minimum lethal dose (MLD) in septic mice was determined by randomly dividing 32 mice into four groups (*n* = 8) and administering intraperitoneal injections of 100 μL of MRPA1 at four different concentrations (5 × 10^8^ CFU/mL, 1 × 10^8^ CFU/mL, 5 × 10^7^ CFU/mL, and 1 × 10^7^ CFU/mL). The clinical signs of the mice were observed within 7 days, and the survival rate of the mice was calculated. MLD is defined as the minimum bacterial suspension concentration causing death in all mice within 24 h, with the value reported as the actual CFU administered per mouse.

To evaluate the therapeutic efficacy of phages in septic mice, 30 mice were randomly divided into three groups (10 mice per group: control group, treatment group 1, and treatment group 2). Each group received an intraperitoneal injection of 100 μL of 5 × 10⁸ CFU/mL MRPA1. One hour later, mice in the control group, treatment group 1 (MOI = 1), and treatment group 2 (MOI = 10) were injected with 100 μL of PBS, 100 μL (5 × 10^8^ PFU/mL) of phage, and 100 μL (5 × 10^9^ PFU/mL) of phage, respectively. The clinical signs of mice in all groups were observed every 24 h for 7 days, and the survival rate was calculated.

To study the pharmacokinetics of phage PaeP_Ls in uninfected mice. Eighteen healthy mice were selected, and each one was injected intraperitoneally with 100 μL (1.0 × 10^9^ PFU/mL) of phage. Three mice were randomly selected for euthanasia at 0.5, 6, 12, 24, 48, and 72 h, and blood was collected by cardiac puncture in an anticoagulant tube of approximately 1 mL, while 1 g of each of the lungs, livers, and kidneys was aseptically taken to make a tissue homogenate with 1 mL of PBS buffer. The anticoagulated blood and tissue homogenate were centrifuged at 12,000 *g* for 5 min, and the supernatant was filtered through a sterile 0.22 μm filter and then diluted doubly, and the phage potency was detected by double-layer plate assay. The number of active phages in the peripheral blood and organs of mice was expressed as PFU per milliliter or PFU per gram.

In order to detect the bacterial load and phage content in the blood of mice, 40 BALB/c mice were randomly divided into two groups: control group (100 μL 5 × 10^7^ CFU/mL MRPA1 bacteria + 100 µL SM buffer) and treatment group (100 μL 5 × 10^7^CFU/mL MRPA1 bacteria + 100 µL 5 × 10^8^PFU/mL phage after 1 h). Three randomly selected animals were euthanized at 6, 12, 24, 48, and 72 h after injection in the treatment and control groups, respectively, and 100 μL of blood was collected by cardiac puncture in EDTA-anticoagulated microcentrifuge tubes and then diluted in multiplicity to measure the bacterial concentration (Log CFU/mL) and phage potency (Log PFU/mL).

For histopathologic studies, 40 BALB/c mice, randomly divided into four groups, were injected intraperitoneally in each group: group A (100 μL of PBS + 100 μL of SM buffer after 1 h), group B (100 μL of 5 × 10^9^ PFU/mL of phage 1 h prior to infection + 100 μL of MRPA1 bacterial solution after 1 h), group C (100 μL of MRPA1 bacterial solution + 100 μL of SM buffer) and group D (100 μL MRPA1 bacterial solution + 100 μL 5 × 10^9^ PFU/mL of phage after 1 h). The mice were killed 24 h after the inoculation, and the liver, spleen, lung, and kidney tissues of five mice in each group were dissected and extracted, soaked in 4% paraformaldehyde, and sent to Sichuan Sainty for pathologic sectioning. Mice were euthanized by cervical dislocation under isoflurane anesthesia to minimize suffering. After hematoxylin and eosin staining, the tissues were scanned on a HISTECH Whole-Section Imaging System to record the pathology of the liver, lung, and kidney of the mice.

### Statistical analysis

All data were plotted using GraphPad Prism 8.0 software. Survival curves were analyzed using the Kaplan-Meier method, and the Log-rank test was used for analysis. All other data were expressed as mean ± standard deviation (SD). Differences between groups were tested using the Mann-Whitney *U* test. *P* < 0.05 was considered a statistical difference, and *P* < 0.01 was considered a statistically significant difference.

## RESULTS

### Phage morphology, stability, and host range assay

**Fig 1:**
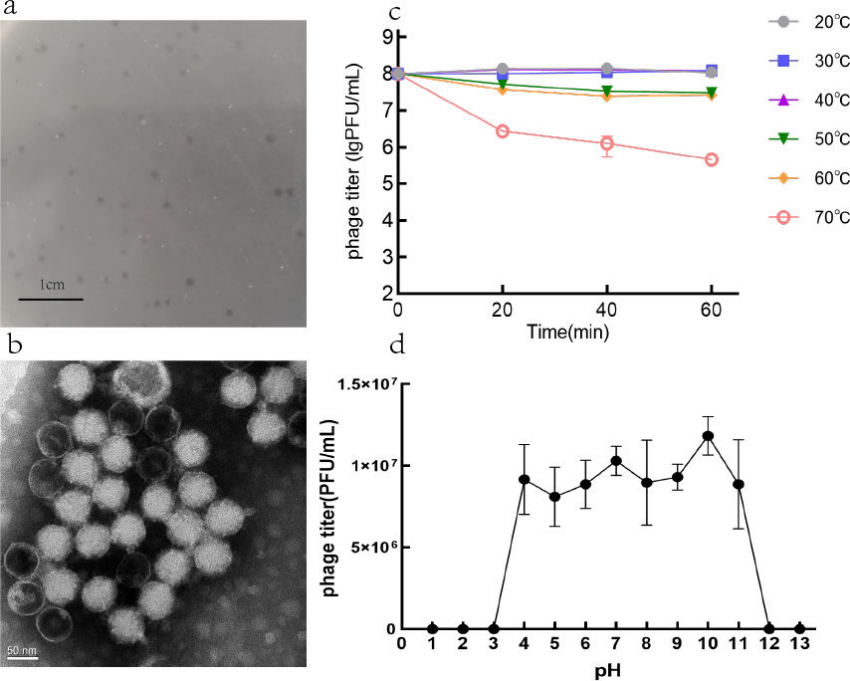
Phage morphology and stability. (**a**) Phage plaques of phage PaeP_Ls. (**b**) TEM image of PaeP_Ls. (**c**) Temperature stability of PaeP_Ls phage (phage titer was determined by sampling after incubating the phage at different temperatures for 20, 40, and 60 min). (**d**) pH stability of PaeP_Ls phage (phage titer measured after exposure to each pH value for 2 h). The results are presented as mean values ± SD.

Phage PaeP_Ls formed a transparent circular plaque with a diameter of 1.1 ± 0.1 mm on MRPA1 (Fig. 1a). TEM showed that it had an icosahedral head with a diameter of 66.7 ± 2.5 nm and a short non-contractable tail with a length of 26 ± 2.0 nm (Fig. 1b). TEM and whole genome sequencing analyses classified PaeP_Ls as members of the family *Autographiviridae* and the genus *Bruynoghevirus*. PaeP_Ls activity was relatively stable at 20°C–60°C (Fig. 1c). In addition, the titer of PaeP_Ls was stable at pH 4–11 but inactivated in strong acid (pH 1–3) or strong base (pH 12–13) environments (Fig. 1d), suggesting that the phage is tolerant to acids and bases.

As shown in Table 1, the host profile of phage PaeP_Ls was determined by spot realizations, indicating that the phage could lyse 53.33% (8/15) of the tested strains.

**TABLE 1:** Host range of PaeP_Ls^*a*^

Strain name	PaeP_Ls sensitivity
MRPA 2	−
MRPA 3	+
MRPA 4	+
MRPA 5	−
MRPA 6	+
MRPA 7	−
MRPA 8	+
MRPA 9	+
MRPA 10	−
MRPA 11	−
MRPA 12	−
MRPA 13	+
MRPA 14	+
MRPA 15	+
MRPA 16	−

^*a*^ “−” = no sensitivity; “+” = sensitivity.

### Biological characterization of PaeP_Ls, *in vitro* bacterial inhibition assays, and altered resistance of resistant strains

**Fig 2:**
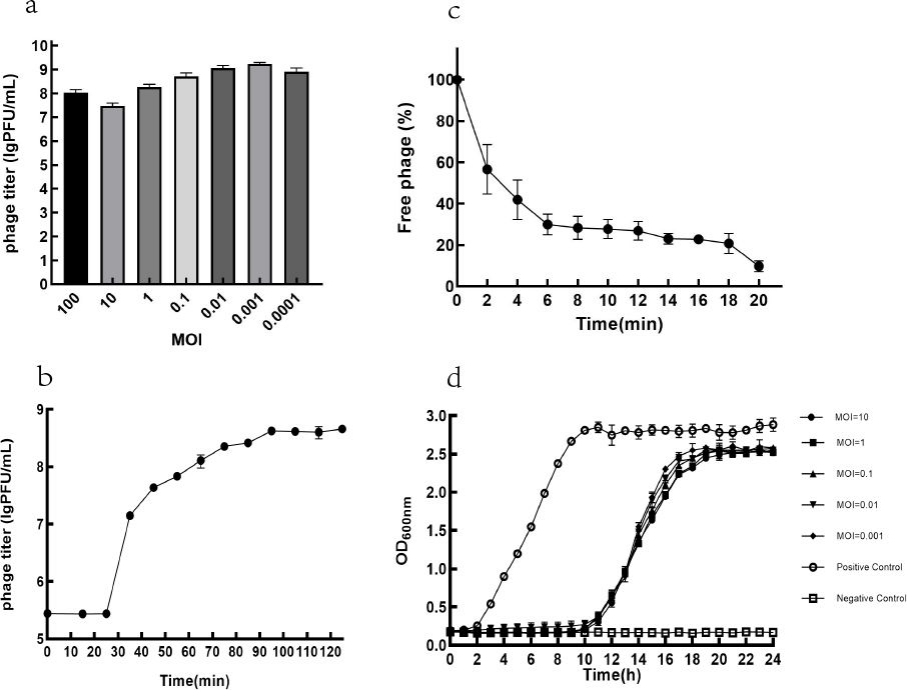
Biological characteristics and *in vitro* antibacterial test of PaeP_Ls. (**a**) Optimal MOI (the optimal MOI was 0.001). (**b**) One-step growth curve (the latent and lysis periods were 25 and 70 min, and the average burst size was about 50 PFU/infected cell). (**c**) Adsorption curve (at 6 min post-infection, approximately 70% of the phages was adsorbed into the host bacteria). (**d**) *In vitro* bacteriostatic experiment of PaeP_Ls under different MOIs (at MOI values of 0.01, 0.1, 1, and 10, the phage effectively inhibits the growth of MRPA1). The results are presented as mean values ± SD.

The highest titer of PaeP_Ls was 1.69 × 10^9^ PFU/mL when the MOI was 0.001 (Fig. 2a), indicating that the optimal MOI of phage PaeP_Ls was 0.001 under our conditions. The adsorption rate of the phage PaeP_Ls is shown in Fig. 2c. At 6 min post-infection, approximately 70% of the phages was adsorbed into the host bacteria. The one-step growth curve revealed that the latent and lysis periods were 25 and 70 min, and the average burst size was about 50 PFU/infected cell (Fig. 2b). The *in vitro* inhibition curve of PaeP_Ls showed that the OD_600 nm_ values of the experimental group were significantly lower than those of the positive control group. However, we observe the re-growth of the population after 11 h, which may indicate the evolution and emergence of phage-resistance strains (Fig. 2d).

The phage resistance mutant R-MRPA1 showed a decrease in MIC value from 32 to 16 against amitrazine (Table 2), but the susceptibility to the other tested antibiotics was unchanged. Since only one resistant mutant was analyzed, it is not possible to draw general conclusions, but the trend deserves to be explored in depth in future studies utilizing more replicated samples.

**TABLE 2:** Drug sensitivity results of MRPA1 and R-MRPA1^*a*^

Antibiotics	MRPA1 (MIC)	Results	R-MRPA1 (MIC)	Results
Ticarcillin/clavulanic acid	≥128	R	≥128	R
Piperacillin/tazobactam	16	S	16	S
Ceftazidime	2	S	2	S
Cefperazone/sulbactam	32	I	32	I
Cefepime	8	S	8	S
Aztreonam	32	R	16	I
Imipenem	≥16	R	≥16	R
Meropenem	≥16	R	≥16	R
Amikacin	8	S	8	S
Tobramycin	≤1	S	≤1	S
Ciprofloxacin	≥4	R	≥4	R
Levofloxacin	≥8	R	≥8	R
Doxycycline hydrochloride	≥16	R	≥16	R
Minocycline	≥16	R	≥16	R
Tigecycline	≥8	R	≥8	R
Polymyxin B	≤0.5	S	≤0.5	S
Compound sulfamethoxazole	≥320	R	≥320	R

^*a*^ R, resistance; I, intermediate; S, susceptible.

### Genomic analysis of phage PaeP_Ls

**Fig 3:**
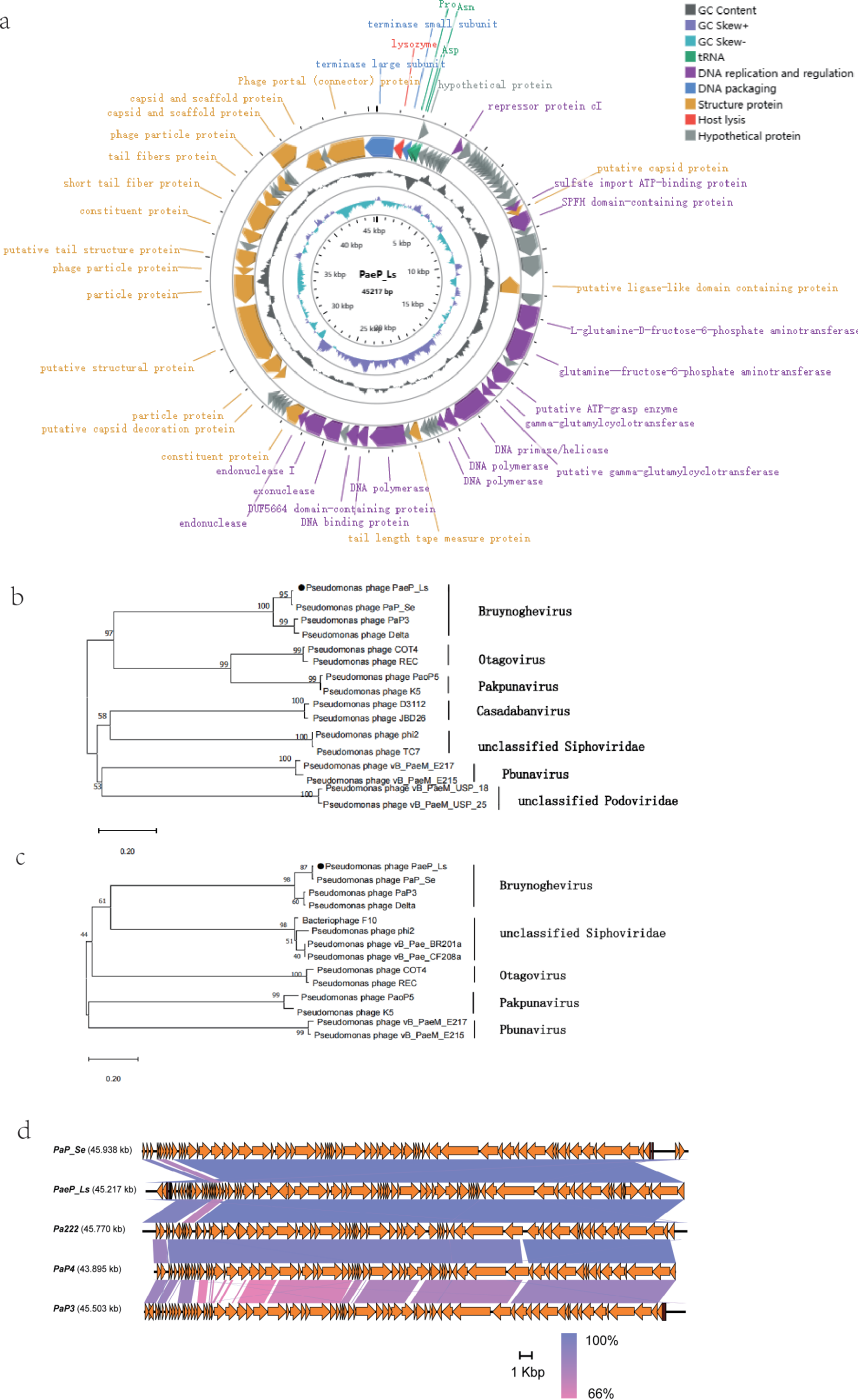
Genomic analysis of phage PaeP_Ls. (**a**) Genome map of phage PaeP_Ls. Nucleotide sequences were analyzed using RAST and the online BLAST. Arrows represent the predicted CDS. Assigned functions are as follows: DNA replication and regulation (purple), DNA packaging (blue), structural protein (yellow), host lysis (red), and a hypothetical protein (gray) (color figure online). Neighbor-joining phylogenetic trees for the amino acid sequences of (**b**) terminase large subunit and (**c**) tail fiber protein showing the relationships between phage PaeP_Ls and other known phages. (**d**) Comparison of the PaeP_Ls genome with PaeP_Se, Pa222, PaP4, and PaP3, the type phage of the Bruynoghevirus genus. This figure was created using Easyfig 2.2.3. The orange arrows indicate CDS, purple lines indicate higher similarity, and pink lines indicate lower similarity.

Figure 3a shows the genome map of PaeP_Ls. It was a double-stranded DNA (dsDNA) virus with a genome size of 45,217 bp and GC content of 52.46% (GenBank accession number: OP342787). The four bases of PaeP_Ls content included A = 24.17%, G = 26.46%, T = 23.37%, and C = 26.01%. The presence of tRNAs results in different levels of A and T, G and C in dsDNA. Three tRNAs, tRNA-proline (Pro), asparagine (Asn), and aspartic acid (Asp) were detected in the PaeP_Ls genome. No known drug resistance genes or virulence factors were predicted in the PaeP_Ls genome, indicating that the phage is relatively safe. We identified a total of 398 open reading frames (ORFs) larger than 150 bp in the PaeP_Ls genome using the ORF finder software, of which 203 and 195 ORFs were located in the positive and reverse strands, respectively. Among all the ORFs of PaeP_Ls, 78 ORFs could be encoded as CDSs. In particular, 68 CDSs (87.18%) had an ATG start codon, 4 had a GTG start codon, and 6 carried a TTG start codon. A total of 37 (47%) of the 78 CDSs encoded functional proteins, and the remaining proteins with unpredicted functions were named hypothetical proteins. Subsequently, all forecasted 37 CDSs were classified into four functional groupings: (i) DNA replication and regulation including 17 CDSs (e.g., repressor protein CI, SPFH domain-containing, DNA primase/helicase, DNA polymerase, DNA binding protein, and exonuclease); (ii) structural proteins including 17 CDSs (e.g., putative capsid protein, tail fiber protein, and capsid and scaffold protein); (iii) DNA packaging, including two CDSs (e.g., terminase large subunit and terminase small subunit); and (iv) host lysis, including lysozyme (Fig. 3a).

We used the neighbor-joining method to analyze the phylogeny of PaeP_Ls to investigate the evolutionary position of PaeP_Ls. The phage terminase large subunit and tail fiber protein are commonly selected for phage phylogenetic analysis (27, 28). Therefore, we selected terminase large subunit (CDS1) and tail fiber protein (CDS69) to construct a phylogenetic tree, which indicated a close relation between Pseudomonas phages PaP_Se, PaP3, Delta, and PaeP_Ls (Fig. 3b). Among them, PaeP_Ls was in the same branch as PaP_Se, indicating its closest parentage to PaP_Se (Fig. 3c). This was consistent with BLAST results with nucleotide similarity (identity 98.51% and query cover 97%) to PaP_Se (Fig. 3d). We concluded that phage PaeP_Ls is a member of the genus Bruynoghevirus from the family and is also related to the genera Otagovirus, Pakpunavirus, and Pbunavirus.

### Phage therapy in a mouse sepsis model

#### Survival rate and phage efficacy of *Pseudomonas aeruginosa* sepsis mice

**Fig 4:**
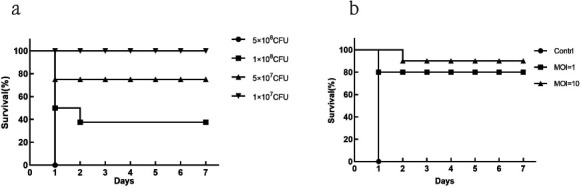
Survival rate and phage efficacy of *Pseudomonas aeruginosa* sepsis mice. (**a**) Survival of mice with different doses of *P. aeruginosa*. The survival rate of the mice was observed and counted for 7 days. (**b**) Analysis of the therapeutic effect of different doses of PaeP_Ls phage. Mice were injected intraperitoneally with 100 μL PBS, 100 μL of 5 × 10^8^ PFU/mL (MOI = 1) phage, and 100 μL of 5 × 10^9^ PFU/mL (MOI = 10) after 1 h of infection.

Mice were divided into four groups (*n* = 8) and then injected intraperitoneally with 100 μL of 5 × 10^8^, 1 × 10^8^, 5 × 10^7^, and 1 × 10^7^ CFU/mL of MRPA1, and the mortality rates of mice at 24 h were 100%, 50%, 25%, and 0%, respectively (Fig. 4a). Therefore, the MLD determined in this experiment was 5 × 10⁷ CFU per mouse (corresponding to 100 μL of a suspension at a concentration of 5 × 10⁸ CFU/mL). One day after infection with the bacterium, clinical manifestations included reduced diet, decreased activity, and symptoms such as shrugging hair, eye discharge, and difficulties opening eyes. All mice in the control group died, the survival rate of mice with MOI = 1 (5 × 10^8^ PFU/mL) and MOI = 10 (5 × 10^9^ PFU/mL) was 80% (*P* = 0.0004) and 90% (*P* < 0.0001), respectively. The experimental results showed that the survival rate of mice treated with phages at MOI values between 1 and 10 was highly significant compared with that of the PBS control group (Fig. 4b). Therefore, PaeP_Ls could effectively treat MRPA1-induced sepsis in mice. Additionally, we observed a dose-dependent protective trend, with the higher MOI (10) treatment group exhibiting the highest survival rate (90%).

#### Dynamics of phage and bacteria in mice

**Fig 5:**
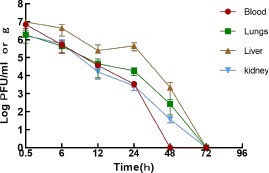
Pharmacokinetics of phage PaeP_Ls in uninfected mice.

As shown in Fig. 5, PaeP_Ls was quickly distributed in lungs, liver, kidneys, and peripheral blood after intraperitoneal injection, and high concentrations of active phage could be detected in the above tissues and peripheral blood at 0.5 h. However, the distribution of the tissues was not homogeneous, with the liver and peripheral blood containing higher levels, and the lungs and kidneys containing less. At 24 h after injection, the concentration of phage PaeP_Ls in all tissues and peripheral blood decreased significantly. At 48 h after injection, the active phage completely disappeared from the peripheral blood, and at 72 h, no active phage existed in all tissues examined in the mice.

**Fig 6:**
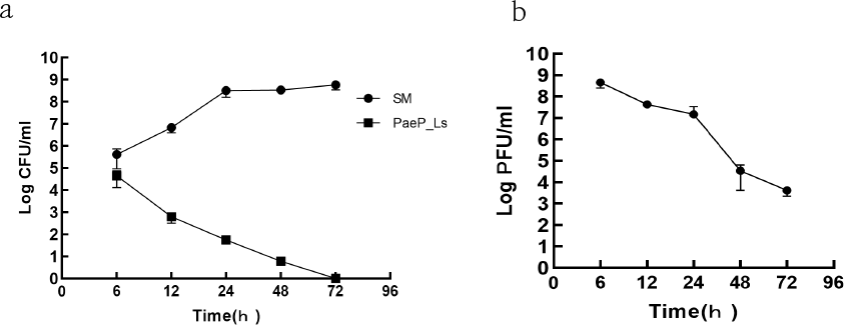
Bacterial and phage levels in the blood of mice. (**a**) Bacteria in the blood of mice. (**b**) Phage in the blood of mice.

In the SM control group, the number of colonies in the blood increased with increasing infection time until the mice died. Compared with the control group, the bacterial load in the blood of the phage-treated group decreased continuously, and no bacteria were detected in the blood at 72 h (Fig. 6a). The phage titer decreased with the decreasing number of colonies in the treatment group (Fig. 6b).

#### Pathological examination

**Fig 7:**
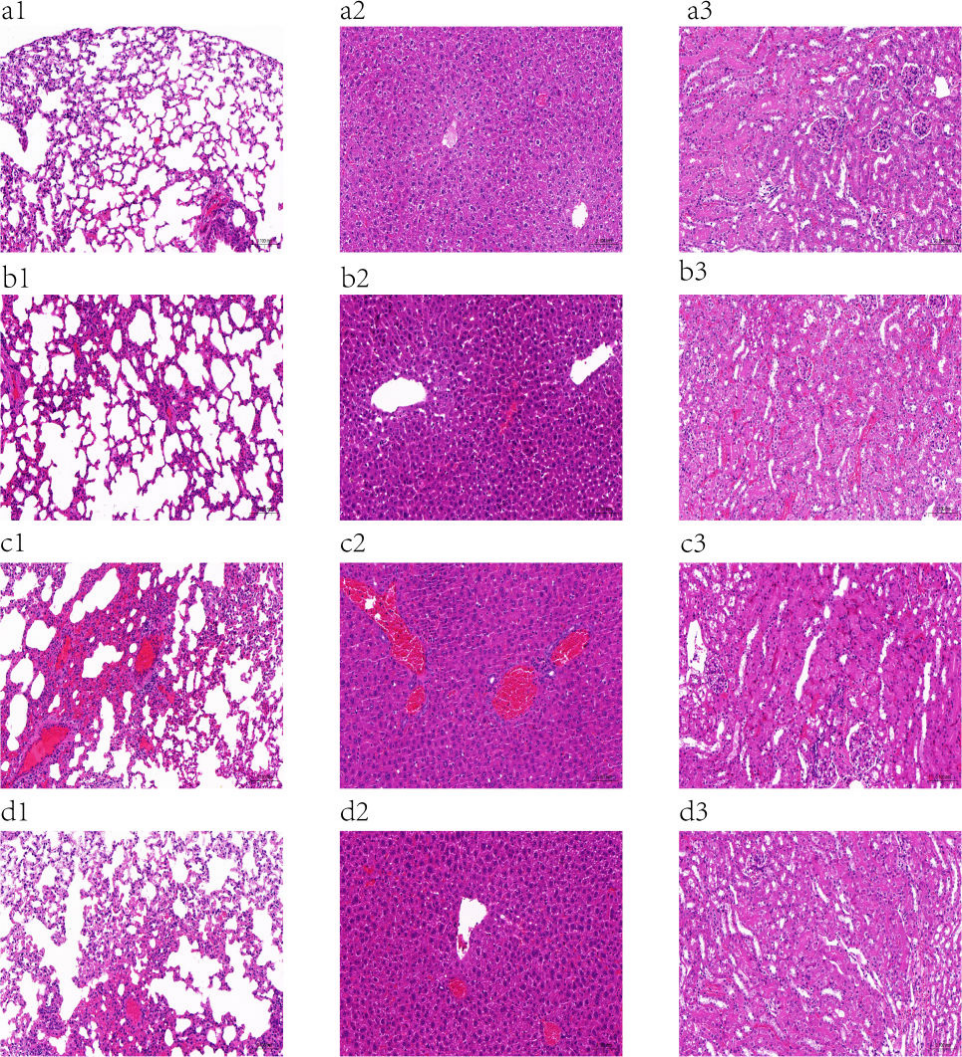
Pathological sections of lungs, liver, and kidneys of mice (200× magnification of the image). (**a**) In the control group, no abnormal histopathological changes were found in tissue sections. (**b**) Phage prevention group, partial necrosis and apoptosis of histiocytes. (**c**) Infection group, massive necrosis and apoptosis of histiocytes. (**d**) In the treatment group, there were only local lesions in the lung, liver, and kidney tissues of mice. 1, 2, and 3 correspond to the lung, liver, and kidney tissues of mice.

Histopathological analysis of mice showed that in Fig. 7, no abnormal histopathological changes were found in the tissue sections of the lung, liver, and kidney of the control group. The normal tissue structure of the lungs, liver, and kidneys of the infected mice disappeared, and congestion, edema, and thickening were obvious, and a large number of inflammatory cells were infiltrated. The degree of lesions in each organ of the mice in the treatment group was mild, with only local lesions, a small amount of congestion, and inflammatory cell infiltration. The lesions of all organs in the prevention group were moderate. It can be seen that the phage has certain therapeutic and preventive effects on sepsis in mice.

## DISCUSSION

In this study, a bacteriophage PaeP_Ls was successfully isolated and characterized from the effluent of our hospital, and then a mouse model of sepsis was established to evaluate the efficacy of this phage in mice. The results show that bacteriophage PaeP_Ls is a promising therapeutic antimicrobial agent, which can not only change bacterial resistance and provide the possibility of antibiotic reuse but also show excellent efficacy in the treatment of carbapenem-resistant *P. aeruginosa*-induced sepsis in mice.

PaeP_Ls was similar to the virulent *P. aeruginosa* phage LUZ24 and manifested as a clear plaque about 1.1 mm in diameter (29). Our study showed that PaeP_Ls remains stable at higher temperatures and within a certain pH range to resist changes in the external environment, facilitating phage transport, storage, and treatment. The latent period and burst size of PaeP_Ls in the MOI (0.001) were 25 min and about 50 PFU/cell, which was different from the latent period of 20 min and burst size of 91 PFU/cell of phage Lx18 (30). The phage’s small burst size may limit future applications. Based on *in vitro* experiments, phage PaeP_Ls has the ability to lyse MRPA1; however, the lysis ability of various MOIs did not differ significantly. At the same time, we observed that the OD600nm value gradually increased after 11 h of co-culture with the host bacteria, which is due to the evolution of phage-resistant strains. It is not uncommon for phage-resistant mutants to emerge rapidly in such a short period, as has been seen in the previously reported phages φVPE25 (31), phage NK5 (32), and GH-K1 (33). Studies have shown that phage-resistant mutants are found in 50% of studies on sepsis models and 3/4 of clinical trials, which is also one of the main problems with phage therapy (13). Numerous studies suggest that phage-resistant mutants may restore bacterial susceptibility to antibiotics (34, 35), thereby extending the lifespan of existing antimicrobials and thus producing positive results for treatment. In addition, in the single phage-resistant mutant we obtained, we observed a slight decrease in the MIC value for amitranam, and although this finding lacks statistical validity, we can follow up with more experiments to verify whether phages can re-sensitize bacteria to antibiotics, thus offering the possibility of antibiotic reuse.

The whole genome sequence of PaeP_Ls shows that the genome is a 45,217 bp long dsDNA sequence with 78 CDS. The genome does not carry any harmful genes, such as genes related to lysogenicity, antibiotic resistance, or toxins. This suggests that PaeP_Ls is a potentially therapeutic MRPA virulent phage. Notably, PaeP_Ls has three different DNA polymerase genes (i.e., CDS42, CDS43, and CDS50). The PaeP_Ls and PaP3 genomes have similar structural features, with the first DNA polymerase gene closely following the DNA primase/helicase genes (36), which overlap by 16 bp in the PaeP_Ls and by 37 bp in the PaP3 genome. These three steps are intimately linked during DNA replication: a helicase unwinds the duplex DNA, the primase catalyzes the formation of short RNA primers, and finally, DNA polymerase catalyzes DNA synthesis in the presence of primers (37, 38). Therefore, it is crucial that the DNA polymerase and DNA primase/helicase genes in PaeP_Ls are closely linked.

*In vivo* experiments in mice with sepsis demonstrated that intraperitoneal injection of PaeP_Ls with MOI = 10 significantly increased the survival rate of mice to 90% (*P* < 0.0001). This is similar to MRPA SaPL, whose survival rate in the treated group of mice treated for MRPA bacteremia was 100%, which was statistically significant (*P* < 0.05) compared to the survival rate of mice in the untreated group (0%) (39). There are several reports that *P. aeruginosa* phage can reduce bacterial load and damage in various tissues (40–42). In this study, phage PaeP_Ls reduced bacterial load in the blood, attenuated histopathological damage, and inflammatory cell infiltration in mice. These results suggest that PaeP_Ls could be used as an alternative approach for the treatment and prevention of MRPA1 infections. The antimicrobial activity of phage *in vivo* depends mainly on the number of active phages, with the immune system being the main factor influencing phage pharmacokinetics, including the intrinsic immune system (e.g., phagocytes, natural antibodies, and complement), which is the first to clear exogenous phages, and acquired immunity (phage-specific antibodies), which is built up in response to phage antigens. Among these, phagocytes are abundantly present in the spleen and liver and have been shown to be the main “phage trap” *in vivo* (43, 44). PaeP_Ls had higher titers in the liver at 24 h post-injection. This is similar to the results for *P. aeruginosa* phage øPEV20, which accumulated significantly in the spleen and liver of rats after intravenous administration (45).

This study focuses on evaluating the efficacy of a single high-dose intraperitoneal injection of phage in septic mice, and there is still a need to evaluate how repeated phage administration, as well as in models of chronic infection, can improve the efficacy of phage without overloading the host immune system. While this study focused on evaluating PaeP_Ls as a single-agent therapy, we acknowledge that phage cocktails or antibiotic-phage combinations may mitigate resistance emergence. Our *in vitro* findings suggest potential synergies for future exploration. The changes in antibiotic sensitivity observed *in vitro* require validation using bacterial isolates from treated animals. Such studies will better reflect clinical scenarios and are planned as a follow-up to this work.

### Conclusion

We provide evidence of successful phage therapy using lysed phages isolated from wastewater against an MRPA-infected mouse model.

## Data Availability

The data presented in this study can be found in the GenBank database, accession number OP342787.
